# Practical Issues in Evidence-Based Use of Performance Supplements: Supplement Interactions, Repeated Use and Individual Responses

**DOI:** 10.1007/s40279-017-0687-1

**Published:** 2017-03-22

**Authors:** Louise M. Burke

**Affiliations:** 1 Sports Nutrition, Australian Institute of Sport, PO Box 176, Belconnen, ACT 2616 Australia; 2 Mary MacKillop Institute for Health Research, Australian Catholic University, Melbourne, VIC Australia

## Abstract

Current sports nutrition guidelines recommend that athletes only take supplements following an evidence-based analysis of their value in supporting training outcomes or competition performance in their specific event. While there is sound evidence to support the use of a few performance supplements under specific scenarios (creatine, beta-alanine, bicarbonate, caffeine, nitrate/beetroot juice and, perhaps, phosphate), there is a lack of information around several issues needed to guide the practical use of these products in competitive sport. First, there is limited knowledge around the strategy of combining the intake of several products in events in which performance benefits are seen with each product in isolation. The range in findings from studies involving combined use of different combinations of two supplements makes it difficult to derive a general conclusion, with both the limitations of individual studies and the type of sporting event to which the supplements are applied influencing the potential for additive, neutral or counteractive outcomes. The repeated use of the same supplement in sports involving two or more events within a 24-h period is of additional interest, but has received even less attention. Finally, the potential for individual athletes to respond differently, in direction and magnitude, to the use of a supplement seems real, but is hard to distinguish from normal day to day variability in performance. Strategies that can be used in research or practice to identify whether individual differences are robust include repeat trials, and the collection of data on physiological or genetic mechanisms underpinning outcomes.

## Introduction

According to surveys and the experience of most sports nutrition professionals, there is a high prevalence of use of sports foods and supplements among competitive athletes [1]. While there are some concerns associated with such observations, particularly around the indiscriminate use of performance supplements [2], many expert groups now take a pragmatic approach to the use of products and protocols which have passed a risk:benefit analysis of being safe, effective, and legal, while also being appropriate to the athlete’s age and maturation in their sport [2, 3]. Indeed, a number of supplements have received plentiful and insightful attention from sports scientists to produce robust evidence of the scenarios in which they can enhance sports performance. These include caffeine [4, 5], creatine monohydrate [6, 7], bicarbonate [8, 9], beta-alanine [10, 11], and beetroot juice/nitrate [12, 13].

While the evidence-base for the use of these products is generally sound, it typically produces a recommended protocol that is generic and isolated from other performance strategies that the athlete may also be implementing. Indeed, the laboratory situations in which most supplement studies are conducted often fail to include many of the important features associated with competitive sport. Earlier work identified a range of characteristics which should be included in investigations of strategies to enhance sports performance to allow them to be more easily applied to competitive athletes [14, 15]. These included using highly trained participants to whom the results are intended to apply, choosing performance protocols which mimic real-life sport, and incorporating other nutritional strategies or features according to the way they should be practiced in the targeted sport or event. Such features have been integrated into many recent investigations, including a number of field studies in which the effect of a performance supplement has been observed during simulated [16, 17] or actual [18] sporting events.

Despite these advances, there are several real-life issues related to the use of performance supplements that remain relatively ignored. Poor interrogation of such issues makes it difficult for athletes to undertake a thorough risk:benefit analysis of their potential use of a supplement or to implement a scenario-specific protocol for their product use that is truly evidence-based. These issues included the additive and interactive effects of combining the use of several performance supplements for a single event, considerations regarding the repeated use of a performance supplement within a relatively brief period, and the notion of individual responsiveness to supplement use. The aim of this review is to examine our current state of knowledge around these issues. It will focus on the performance supplements which were previously identified as enjoying strong support for their benefits to the performance of a single competitive event. In addition, phosphate will be considered in view of the emerging information about its potential benefits to sports performance [19] as well as the existence of several studies of its combined use with other evidence-based supplements. Protocols of use and mechanisms of action of these supplements are summarized in Table 1 to provide a backdrop to these discussions.

**Table 1:** Summary of evidence-based uses of performance supplements to benefit competition outcomes

Supplement	Mechanism of action	Recommended protocol of use	Targeted events
Bicarbonate [8, 9]	Temporarily increases blood bicarbonate to acutely enhance extra-cellular buffering of efflux of H+ ion from contracting muscle. Reduces fatigue associated with exercise in which there is production of large amounts of H^+^ ions via anaerobic glycolysis	300 mg/kg bicarbonate (as sodium bicarbonate) Split doses @ 2–2.5 h pre-event	Sustained high-intensity events lasting 2–8 min (e.g., swimming, rowing, track cycling and athletics) Intermittent high-intensity sports with prolonged efforts above lactate threshold (e.g., team sports, combat sports) Sustained events just below lactate threshold which involve higher-intensity efforts (e.g., cycling time trials, 5000–1000 m running)
Beta-alanine [10, 11]	Increases muscle carnosine to chronically enhance intra-cellular buffering and buffering of the efflux of H^+^ ion from contracting muscle. Reduces fatigue associated with exercise where there is production of large amounts of H^+^ ions via anaerobic glycolysis. May have other roles in cell	Loading: 200+ g beta-alanine taken over 4–10 weeks in split daily doses (e.g., 3 × 3.2 g/day for 4 w or 2 × 2.4 for 10 w) Maintenance: ~2 g/day	Acute/competition performance Sustained high-intensity events lasting 1–8 min (e.g., swimming, rowing, track cycling and athletics) Chronic enhancement of high intensity training may have long-term benefit to other events
Caffeine [4, 5]	Multiple actions but most important ones are likely to be a reduction in perception of effort or pain or a direct effect on muscle contraction	3–6 mg/kg caffeine (total dose) Numerous protocols involving single or serial intake, and including pre-event intake, and in longer events, intake during and at the end of the event	Sustained high-intensity events lasting 1–8 min (e.g., swimming, rowing, track cycling and athletics) Sustained high-intensity events of 10–60 min Intermittent high-intensity sports (e.g., team sports on field and on court, racket sports, combat sports) Endurance (e.g., marathon, cross-country skiing) and ultra-endurance (e.g., Ironman, cycling road races) Prolonged skill sports (e.g., golf, shooting)
Creatine [6, 7]	Increased PCr content enhancing capacity for repeated bouts of high-intensity exercise with brief recovery intervals which would otherwise provide inadequate recovery of PCr stores. May have other roles in upregulating cell protein synthesis	Loading: 5 days @ 20 g/day in split doses or 28 days @ 2–3 g/day as creatine monohydrate Maintenance: 2–3 g/day	Acute/competition performance Intermittent high-intensity sports (e.g., team sports, racket sports, combat sports) Chronic enhancement of interval and resistance training may have long-term benefit to other events
Nitrate/beetroot juice [12, 13]	Enhanced production of nitric oxide via oxygen-independent pathway from nitrate increases exercise efficiency/economy and enhances exercise capacity.	~8 mmol nitrate in beetroot juice (or as sodium nitrate) taken 2–2.5 h pre-event May be accentuated by pre-loading with this dose for 3–5 days pre-event	Higher-intensity events of up to 4–8 min (e.g., track cycling, athletics, rowing and swimming events) Events involving local or systemic acidosis/hypoxia (e.g., swimming, rowing, and other upper-body sports) Athletes of lower training status/caliber
Phosphate [19]	Range of mechanisms including increased buffering capacity, increased 2,3-diphosphoglycerate to increase dissociation of O_2_ into muscles and increase phosphate availability for ATP synthesis	3–6 days @ 3–5 g/day as sodium phosphate	Less certainty over events that may benefit from phosphate supplementation but may include High intensity events of 2–8 min Endurance events Intermittent high-intensity sports such as team sports

*PCr* phosphocreatine, *ATP* adenosine triphosphate

## Additive and Interactive Effects of the Use of Combinations of Performance Supplements

Many studies of supplementation practices in sport note that athletes report the simultaneous use of a number of different products [20–22]. Undoubtedly, some athletes practice indiscriminate polypharmacy, consuming large numbers of products without apparent consideration of the cumulative quantity and range of ingredients that are ingested [20]. However, the current review focusses on the deliberate combination of several supplements in a competition setting with the goal of optimizing performance benefits via an additive effect or positive interaction. It consciously excludes discussion of the growing number of multi-ingredient performance supplements which involve a blend of ingredients (sometimes up to 30 individual substances) due to a number of concerns. Many multi-ingredient performance supplements represent a practical risk for athletes because of their (sometimes undeclared and large) content of stimulants and/or banned substances [23, 24], failure to provide effective doses of evidence-based ingredients, or failure to disclose ingredients by claiming protection of their “proprietary blend.” As such they are problematic in relation to health, doping safety, and efficacy/value for money [2]. Although studies of the performance effects of commercially available multi-ingredient supplements can be found in the literature [25–27], the results of such investigations are typically confounded by methodological flaws such as comparison to a single placebo or lack of independent verification of the product contents. Since these factors prevent detectable effects from being attributed to a single ingredient or allow interaction between ingredients to be isolated, they cannot contribute to the focus of this review.

Supplements can enhance the outcome of a competitive event if they reduce or delay the onset of the specific physiological factors that would otherwise cause fatigue or decay in performance throughout the event or towards its conclusion. Potential benefits could occur via mechanisms such as increased substrate availability, reduced perception of pain or effort, the buffering of disturbances in cell homeostasis such as changes in muscle pH and/or an increase in the efficiency of muscle contraction. In some sporting events, a number of these conditions or ergogenic opportunities occur, therefore, it is not surprising that several different supplements may be of benefit when used in isolation (see Table 1). The use of a combination of supplements for a single event can occur in several ways: the simultaneous use of several products with individual benefits in the acute scenario of the event, or the acute use of supplements in conjunction with a chronically applied supplement used to support training outcomes. A range of possible outcomes of the addition and interaction of supplements can be identified (see Table 2).

**Table 2:** Considerations for combined or repeated use of performance supplements for a sporting event

Question	Potential outcomes	Theoretical examples
What is the interaction between supplements which are used in combination for a single event?	Related mechanisms with additive benefits	Loading strategies for bicarbonate (extracellular buffer) and beta-alanine (intracellular buffer) may combine to increase total buffering capacity and tolerance of acidosis due to high rates of energy production from anaerobic glycolysis. The combination may be better than either product used in isolation due to the greater increase in buffering capacity per se, as well as opportunity to buffer both within the muscle cell as well promote the efflux of H^+^ from the cell. May be useful for an event/athlete in which substantial drops in muscle pH are experienced
	Related mechanisms with no additive benefits	The benefits of combining bicarbonate and beta-alanine loading protocols may not be evident if the additional buffering capacity provided by either supplement alone is sufficient to address the physiological limitations of the event/athlete
	Independent mechanisms with additive benefits	The benefits of creatine loading (providing additional muscle phosphocreatine substrate) and caffeine (reducing the perception of effort) may combine in a sport involving prolonged repeated efforts. In this case, some other factor is causing fatigue towards the end of the event and caffeine is able to reduce/delay the onset of this effect
	Independent mechanisms with no additive benefits	The benefits of creatine loading (providing additional muscle phosphocreatine substrate) and caffeine (reducing the perception of effort) may not be additive in an event in which fatigue is related to inadequate recovery of phosphocreatine store between sprints. This limitation can be masked by caffeine or addressed by creatine loading. However, once it is addressed, the effect of caffeine is no longer beneficial
	Independent mechanisms with counteractive outcomes (direct)	Nitrate supplementation (nitric oxide production via a separate pathway that can operate in hypoxic and acidic conditions) may enhance the performance of sustained high intensity exercise. However, the addition of bicarbonate loading may reduce the effectiveness of nitrate supplementation by buffering plasma acidosis and removing the conditions where it is valuable
	Independent mechanisms with counteractive outcomes (indirect)	Bicarbonate loading (extracellular buffer) and caffeine (reduced perception of effort) may each enhance the performance of sustained high intensity exercise. However, the co-ingestion of these two supplements may increase the risk of gut side-effects associated with bicarbonate supplementation and may impair performance
What are the potential issues in repeating the use of the same supplement for a subsequent event?	Subsequent use of the supplement might require a different protocol to restore the physiological advantage or to meet the logistical requirements of competition spacing	If the half-life of the supplement is prolonged in relation to the gap between competition events, it may not be necessary to take a complete dose for the subsequent event to achieve its physiological role. For example, smaller doses of bicarbonate, nitrate or caffeine may be suitable as a “top up” for events held 2**–**4 h apart. Alternately, reorganisation of the recognized protocol may be needed if the gap between events is <2 h (and smaller than the suggested interval for supplement intake for nitrate or bicarbonate)
	Desensitization of physiological systems may render the subsequent use of a supplement less effective	The response to some supplements may be reduced by repeat exposure due to a desensitization effect. For example, it has been suggested (probably erroneously) that the performance effects of caffeine are reduced in habitual users and that its use for competition purposes should follow a caffeine withdrawal
	Residual fatigue left from enhanced performance in the first event may carry over to the subsequent event	It is possible that the greater physiological effort made possible by supplementation in the first event may cause residual fatigue that requires management for the second event. For example, use of caffeine in the first event may mask fatigue and allow a higher intensity/power output that may cause acidosis or greater depletion of fuel substrates

The studies located for this review were sourced via a thorough literature search of Medline and Google Scholar databases, as well as the reference lists from the sourced papers. Search terms for the database search were “supplement,” “caffeine,” beta-alanine,” “bicarbonate,” “creatine,” “nitrate,” “beetroot juice,” “phosphate,” “sport,” “performance,” “combination,” “interaction,” and “additive.” Papers were included in the featured analysis (Table 3) if they met each of the following three criteria: recruitment of athletes undertaking specific training for a sport/event, the single and combined application of the selected performance, and implementation of protocols that are applicable to the conduct and outcomes of a sporting event. Due to the scarcity of studies, some lenience was shown in the case of the performance protocol. Table 3 summarizes the available studies that met these criteria and includes information about the specific implementation and outcomes of the supplementation protocols as well as an indication of any apparent interactions.

**Table 3:** Summary of studies of evidence-based performance supplements used singly and in combination

Study	Subjects and design^a^	Dose	Performance measure	Performance benefit	Summary
Bicarbonate and beta-alanine					
Tobias et al. [28]	Well-trained judo and jiu-jitsu athletes (*n* = 37 M) Parallel group design (*n* = 9**–**10) for placebo, bicarbonate, beta-alanine, and combined	7 days @ 500 mg/kg/day sodium bicarbonate split into 4 doses) and/or 28 days @ 6.4 g/day beta-alanine: Total = 179 g	Combat sports simulation 4 × 30 s upper body Wingate tests with 3 min recovery Tests conducted pre and post 28 days of beta-alanine/placebo	Bicarbonate: Yes Beta-alanine: Yes Interaction: related mechanism with additive effects	Compared to placebo, beta-alanine (+7% *p* = 0.003) and bicarbonate (+8% *p* = 0.002) enhanced total work combined bicarbonate and beta-alanine resulted in the greatest gains (+14%, *p* < 0.0001), and significantly reduced perceived exertion. Authors noted that upper body exercise (smaller muscle mass) produces greater H+ disturbances than lower body exercise accounting for clear and additive effects of both buffering systems
Ducker et al. [29]	Competitive team sport athletes (*n* = 24 M) Parallel group design (*n* = 6 M) for placebo, bicarbonate, beta-alanine, and combined	300 mg/kg sodium bicarbonate @ 60 min pre-exercise and/or 28 days @ 80 mg/kg BM/day beta-alanine: Total ~ 168 g	Team sport simulation 3 sets @ 6 × 20 m sprints on 25 s with 45-s recovery Tests conducted pre and post 28 days of beta-alanine/placebo	Bicarbonate: Perhaps Beta-alanine: No Interaction: related mechanism but possible counteractive effect	Effect size comparison revealed that beta-alanine supplementation by itself produced trivial effects on total sprint time, mean sprint time, first sprint and fastest sprint. Whereas bicarbonate alone was associated with “very likely” improvements in these factors, combined beta-alanine and bicarbonate reduced the size of this improvement to “likely”. Small sample sizes were noted as a limitation of this study
De Salles Painelli et al. [30]	Well-trained junior swimmers (*n* = 6 M, 7 F) Crossover design (bicarbonate or placebo) undertaken post parallel group design (beta-alanine or placebo)	300 mg sodium bicarbonate @ 90 min pre-exercise (first swim) and/or 1 w @ 3.2 g/day + 3.5 w @ 6.4 g/day beta-alanine: Total = 202 g	Swimming 100 m 200 m 30-min recovery Tests conducted pre and post 28 days of beta-alanine/placebo. Competition elements built into research design (warm-up, racing against others etc.)	200 m Bicarbonate: Yes Beta-alanine: Yes 100 m Bicarbonate: Yes Beta-alanine: Possibly Interaction: related mechanism with possibly additive effects	Magnitude based inference analysis and conventional statistics showed for 200 m improvements of 2.3, 1.5, and 2.1% for bicarbonate, beta-alanine and combined (*p* < 0.05) from baseline with no change in placebo group. Although combined was NS different to bicarbonate alone, probability of positive effect was 78% 100-m swim showed significant improvement with bicarbonate and combined only. However, probability of beta-alanine having positive effect was 65% alone and 72% when combined. Effects more apparent in longer swim (greater acidosis?)
Mero et al. [31]	International and national swimmers (*n* = 13 M) Crossover design (bicarbonate) before and after all subjects supplemented with beta-alanine	300 mg/kg sodium bicarbonate @ 60 min pre-exercise (first swim) and 28 days @ 4.8 g/day beta-alanine: Total = 134 g	Swimming 2 × 100 m 12 min recovery Tests conducted pre and post 28 days of beta-alanine Competition elements built into research design (warm-up, racing against others etc.)	Bicarbonate: Possibly Beta-alanine: No Interaction: related mechanism but no additive effect	Prior to beta-alanine supplementation, there was no effect of bicarbonate supplementation on swim 1, but there was less attenuation of performance in swim 2 such that performance was ~1.5 s or 2.4% faster (*p* < 0.05). Following beta-alanine supplementation, bicarbonate failed to provide any change in performance of swim 1 or 2. Note that beta-alanine supplementation undertaken without placebo control and with order effect
Hobson et al. [32]	Well-trained male rowers (*N* = 20 M) Crossover design (bicarbonate or placebo) undertaken post parallel group design (beta-alanine or placebo)	300 mg/kg sodium bicarbonate: 200 mg/kg @ 4 h pre-exercise + 100 mg/kg BM @ 120 min pre-exercise and/or 30 days @ 6.4 g/day beta-alanine: Total = 192 g	Rowing 2000 m ergometer TT Tests conducted pre and post 28 days of beta-alanine/placebo	Bicarbonate: Possibly Beta-alanine: Probably Interaction: related mechanism with possible additive effect	Magnitude based inference analysis showed that bicarbonate had a likely benefit on rowing performance (3.2 ± 8.8 s improvement > placebo), whereas beta-alanine was very likely to be beneficial to 2000-m rowing performance (6.4 ± 8.1 s vs. with placebo) and there was a small (1.1 ± 5.6 s) but possibly beneficial additional effect with combined beta-alanine + bicarbonate supplementation vs. with beta-alanine alone
Bellinger et al. [33]	Highly trained cyclists (*N* = 14 M) Crossover design (bicarbonate or placebo) undertaken post parallel group design (beta-alanine or placebo)	300 mg/kg sodium bicarbonate @ 90 min pre-exercise and/or 28d @ 65 mg/kg/day beta- alanine: Total = 129 g	Cycling 4 min ergometer TT Tests conducted pre and post 28 day of beta-alanine/placebo	Bicarbonate: Yes Beta-alanine: No Interaction: related mechanism but no additive effect	Average power output was significantly (*p* < 0.05) increased compared to baseline for bicarbonate (+3.1 ± 1.8%) and combined beta-alanine + bicarbonate (3.3% ± 3.0%), but not for beta-alanine alone (1.6% ± 1.7%) or placebo. Magnitude based inferences noted a 37% probability of benefits from beta-alanine. Some subjects experienced mild paresthesia (side effect of beta-alanine) which may have compromised study blinding
Bicarbonate and caffeine					
Felippe et al. [34]	Regional and national level judo players (*n* = 10 M) Crossover design to produce bicarbonate, caffeine, combined, and placebo trials	300 mg/kg sodium bicarbonate @ 60-120 min pre-exercise and/or 6 mg/kg caffeine @ 60 m pre-exercise	Judo 3 × JSFT interspersed by 5 min	Bicarbonate: Perhaps Caffeine: Perhaps Interaction: independent mechanism with additive effects	Compared with placebo trial, caffeine and bicarbonate trials both produced a small but non-significant increase in the total number of throws performed across all three JSFT tests. However, the combined trial showed a further increase in throws, which reached significance (72.7 ± 3.1 vs. 68.8 ± 4.2 throws, for combined and placebo respectively, *p* = 0.003; ES = 1.05)
Christensen et al. [35]	International level rowers (*n* = 11 M, 1 F; 6 lightweight and 6 heavyweight) Crossover design to produce bicarbonate, caffeine, combined, and placebo trials	300 mg/kg sodium bicarbonate @ 75 min pre-exercise and/or 3 mg/kg caffeine @ 45 m pre-exercise	Rowing 6 min ergometer TT	Bicarbonate: No Caffeine: Yes Interaction: independent mechanism with no additive effect	Compared with placebo trial (1865 ± 104 m), a greater distance was covered with caffeine (1878 ± 97, *p* < 0.05) and combined caffeine/bicarbonate (1877 ± 97, *p* < 0.05) but not for bicarbonate alone (1860 ± 96, *p* = 0.1). Therefore, although bicarbonate was not associated with performance enhancement, neither did it negate the effectiveness of caffeine, as seen in some other studies. Effects more detectable in lightweight rowers
Kilding et al. [36]	Well-trained cyclists (*n* = 10 M) Crossover design to produce bicarbonate, caffeine, combined, and placebo trials	300 mg/kg sodium bicarbonate @ 90**–**120 min pre-exercise and/or 3 mg/kg caffeine @ 60 min pre-trial	Cycling 3 km ergometer TT	Bicarbonate: Yes Caffeine: Yes Interaction: independent mechanism with no additive effect	Compared with placebo trial (373 ± 41 W), a greater mean power output was seen with caffeine (381 ± 67, *p* < 0.05), bicarbonate (383 ± 44, *p* < 0.05), and combined caffeine/bicarbonate (382 ± 39, *p* < 0.05). Study unable to discern mechanism of benefit with combined use and whether one supplement negated the effect of the other
Carr et al. [37]	Well-trained rowers (*n* = 6 M, 2F) Crossover design to produce bicarbonate, caffeine, combined, and placebo trials	300 mg/kg sodium bicarbonate @ 90 min pre-exercise and/or 6 mg/kg caffeine @ 30 min pre-exercise	Rowing 2000 m ergometer TT Overnight fasted	Bicarbonate: No Caffeine: Yes Interaction: independent mechanism with counteractive effect (indirect)	Performance was substantially (~2%) enhanced by caffeine (6:40.8 ± 22 min:s) compared with placebo (6:43.8 ± 23) but differences between bicarbonate (6:44.4 ± 23 min:s), combined (6:42.6 ± 22 min:s) and placebo were unclear. GI symptoms associated with bicarbonate caused failure of expected performance due to enhanced buffering, but also counteracted the benefits of caffeine. Note that protocol did not make use of strategies to reduce GI effects of bicarbonate.
Pruscino et al. [38]	Highly trained swimmers (*n* = 6 M) Crossover design to produce bicarbonate, caffeine, combined, and placebo trials	300 mg/kg sodium bicarbonate spread @ 30**–**120 min pre-exercise and/or 6 mg/kg caffeine @ 45 min pre-exercise	Swimming 2 × 200 m 30 min recovery Competition elements built into research design (warm-up, racing against others etc.)	Bicarbonate: Perhaps Caffeine: No, perhaps harm Interaction: independent mechanism with unclear additive effect (indirect)	Differences between trials for absolute times failed to reach statistical significance. However, magnitude based inference analysis showed generally trivial effects apart from caffeine where effects ranged from trivial effect to large harm. bicarbonate showed a smaller reduction in swimming time from Swim 1 to Swim 2 (0.3 ± 0.7% faster) than caffeine or combined trials (*p* < 0.05). Effect was less evident for a single effort. Majority of athletes recorded fastest TT for single and repeat performance from the combination of bicarbonate and caffeine. Authors suggested that caffeine might have benefit for first swim but this had negative carry-over effect on second swim that was overcome by bicarbonate
Caffeine and nitrate/beetroot juice					
Glaister et al. [39]	Well-trained cyclists (*n* = 14 F) Crossover design to produce BJ, caffeine, combined, and placebo trials	7.3 mmol/day nitrate in BJ @ 2.5 h pre-exercise and/or 5 mg/kg BM caffeine @ 1 h pre-exercise	Cycling 20 km ergometer TT	Nitrate: No Caffeine: Yes Interaction: independent mechanism with no additive effect	Caffeine alone and combined resulted in improved ~3% power output (*p* < 0.05) compared with placebo however differences between these trials were not significant. Time to complete the 43.83 km distance was reduced to a similar extent (1.3%, *p* < 0.05) for both the combined and caffeine trials. BJ had no significant positive or negative effect on time to complete the TT. Using an inference based statistical approach. Caffeine would likely (89%) and very likely (99%) enhance TT performance for caffeine and combined, respectively. BJ was unlikely (7%)/very unlikely (1%) to have any positive effect on performance for BJ and combined, respectively
Lane et al. [40]	Well-trained cyclists (*n* = 12 M) Crossover design to produce BJ, caffeine, combined, and placebo trials	2-day pre-load (8.4 mmol/day nitrate in BJ) + acute dose 8.4 mmol nitrate @ 2 h pre-exercise and/or 3 mg/kg BM caffeinated gum @ 40 min pre-exercise	Cycling 43.83 ergometer TT simulating Olympic course Nutritional conditions simulating race practices (carbohydrate pre-event as mouth rinse during TT)	Nitrate: No Caffeine: Yes Interaction: independent mechanism with no additive effect	Caffeine alone improved power output compared with BJ and placebo (205 ± 21 W vs. 194 ± 22 W and 194 ± 25 W, *p* < 0.05), leading to a time improvement of ~45 s. However BJ had minimal effect on performance alone or in combination, and may have slightly reduced the effect of caffeine since the outcome of the combined trial was neither significantly different to placebo or to caffeine
Lane et al. [40]	Well-trained cyclists (*n* = 12 F) Crossover design to produce BJ, caffeine, combined, and placebo trials	2-day pre-load (8.4 mmol/day nitrate in BJ) + acute dose 8.4 mmol nitrate @ 2 h pre-exercise and/or 3 mg/kg BM caffeinated gum @ 40 min pre-exercise	Cycling 29.35 km ergometer TT simulating Olympic course Nutritional conditions simulating race practices (carbohydrate pre-event as mouth rinse during TT)	Nitrate: No Caffeine: Yes Interaction: independent mechanism with no additive effect	Caffeine alone and combined resulted in improved ~3% power output (*p* < 0.05) compared with placebo however there was no significant difference between these two conditions. Time to complete the 29.35-km distance was faster by 0.9 and 1.6% (*p* < 0.05) for the combined and caffeine trials. respectively. BJ had no significant positive or negative effect on time to complete TT. Using an inference based statistical approach, caffeine would likely (88%) improve TT performance and very likely (99%) enhance power output (caffeine vs. lacebo), with a small reduction in this effect when combined with BJ. BJ was very unlikely (0%) to have any positive effect on performance
Nitrate/beetroot Juice and phosphate					
Buck et al. [41]	Recreationally trained team sport athletes (*n* = 13 F) Crossover design to produce BJ, phosphate, combined, and placebo trials	6 mmol nitrate in BJ @ 2 h pre-exercise and/or 6 days @ 50 mg/kg/day FFM sodium phosphate in split doses Washout of 17 days between trials	Team sport 6× 20 s sprints Undertaken @ 0, 30 and 60 around a 4 × 15 min simulated team game circuit	Nitrate: No Phosphate: Yes Interaction: independent mechanism with possibly counteractive effect	Compared with placebo and BJ, phosphate trial produced faster total sprint time for first and second set of sprints and overall sprints (~5% improvement, *p* < 0.05). Phosphate produced fastest sprint in set 1 and overall compared with placebo (~6% faster). Trend to better sprint times with combined compared with placebo (~2% improvement) suggesting some lessening of the effect of phosphate when combined with nitrate. Effects of phosphate seen when fresh as well as fatigued
Caffeine and phosphate					
Kopec et al. [42]	Trained team sport athletes (*n* = 11 M) Crossover design to produce caffeine, phosphate, combined, and placebo trials	6 mg/kg caffeine @ 60 min pre-exercise and/or 6 days @ 50 mg/kg/dayFFM sodium phosphate in split doses Washout of 17 days between trials	Team sport 6 × 20 s sprints Undertaken @ 0, 30, and 60 around a 2 × 30 min simulated team game circuit	Caffeine: No Phosphate: Perhaps Interaction: independent mechanism with possibly counteractive effect	Although results failed to reach statistical significance, effect size and magnitude based analysis revealed that, compared with placebo, phosphate resulted in the fastest times for all sprints with moderate-large effect sizes and “likely” to “very likely” benefits. The beneficial effects of combined were smaller, and the effects of caffeine alone were minimal
Buck et al. [43]	Recreationally trained team sport athletes (*n* = 12 F) Crossover design to produce caffeine), phosphate, combined, and placebo trials	6 mg/kg caffeine @ 60 min pre-exercise and/or 6 days @ 50 mg/kg/day FFM sodium phosphate in split doses Washout of 17 days between trials	Team sport 6 × 20 s sprints Undertaken @ 0, 30, and 60 around a 4 × 15 min simulated team game circuit	Caffeine: No Phosphate: Yes Interaction: independent mechanism with no additive effect	Overall results showed that combined and phosphate alone improved sprint times when fresh (set 1) and fatiguing (set 2 and 3) compared with placebo. Caffeine alone had small effects

*BJ* beetroot juice, *BM* body mass, *ES* effect size, *FFM* fat free mass, *GI* gastrointestinal, *JSPT* judo specific performance test, *M* male, *F* female, *TT* time trial

^a^Randomized, double-blind, placebo-controlled unless noted

Even when the focus is restricted to a few supplements and the specific scenarios in which there is evidence-based justification of their use, a large number of permutations of supplement use combinations can be identified. Therefore it is not surprising that the current literature, which is relatively recent, fails to cover much of the potential interest. Furthermore, the literature presents an ad hoc collection of studies rather than a systematic coverage of key supplement interactions and is limited by the presence of methodological flaws in some studies. Nevertheless, a number of themes can be identified. Almost all of the available studies involved the investigation of the single or combined benefits of two performance supplements. Indeed, a systematic investigation of the interactions between three or more supplements used is understandably challenging, even though Table 1 identifies that such use could be justified in some sports/events. For example, each of the six performance supplements included in this review, when used in isolation, could theoretically provide a benefit to the preparation (beta-alanine, creatine) or performance (caffeine, beta-alanine bicarbonate, nitrate, phosphate) of a 4000-m track cycling pursuit, 400-m swim, or 2000-m rowing race. A sophisticated approach to field testing or research methodology would be needed to identify the optimal protocol for combining the use of some or all of these supplements.

To date, the most commonly studied combination of performance supplements has been bicarbonate and beta-alanine [28–33]; this represents an example of substances that might interact via a related mechanism of action in enhancing the muscle’s ability to buffer high rates of production of hydrogen ions (H+) (Table 1). The available literature, summarized in Table 3, involves variability in the types of athletes, protocols of supplement use, and measures of sporting performance included in investigations. Not surprisingly, there are a variety of observations ranging from neutral and positive effects on performance from each of the supplements individually, and interactions including counteractive, neutral, and additive (see Table 2). The lack of consistency in outcomes can be attributed partially to limitations in study designs including small sample sizes and the further loss of statistical power due to the need for a parallel group application of the chronic protocol of beta-alanine supplementation. However, the benefit of either supplement or their combined use is also likely to be dictated by the type of sporting event and the degree to which performance is limited by excessive acidosis.

Caffeine and bicarbonate have also received some attention as supplements that might be used in tandem for sporting events involving high-intensity exercise [34–38], with the separate effects of reduced perception of effort and enhanced buffering being able to combine to further enhance performance. Again, the literature shows a lack of consistency in findings, with observations of positive, neutral, and possibly negative outcomes from the individual substances, and a range of additive, neutral, and counteractive effects when they are used in combination (Table 3). Although one study involving a sports specific (judo) performance protocol has reported that the small and unclear positive effects of each supplement in isolation could be combined to produce a significant benefit [34], it appears there is also a potential for one product to cancel the need for the other when both are individually valuable. For example, whereas caffeine and bicarbonate supplementation protocols were both successful in improving the performance of a 3-km cycling time trial when used in isolation, there were no further benefits from their combined use [36]. The mechanism of the performance enhancement with the bicarbonate-caffeine trial was not evident in this study so the nature of the interaction of these supplements remains unclear. However, interactions in other studies include a counteractive response of the positive effects of caffeine due to gastrointestinal disturbances arising from bicarbonate use [37], but also a beneficial effect of using bicarbonate to address greater acidosis associated with a faster effort due to caffeine support [38]. Here again, the scarcity of studies makes it difficult to derive a general conclusion, with both the limitations of individual studies and the type of sporting event to which the supplements are applied governing the potential for misunderstanding the literature and drawing context specific outcomes, respectively.

Other supplement combinations that have received some attention in the literature include the pairing of nitrate/beetroot juice with caffeine [39, 40] or phosphate [41]. In each case there has been a failure to see a benefit from the nitrate supplementation but an enhancement by the other performance supplement and a slight [39, 40] to apparent [41] reduction in the benefit of the combined supplementation protocol. While the former may simply be a quirk of the statistical analysis in a small sample size, there may be some interaction that requires further investigation. Similar findings regarding the combination of caffeine and phosphate [42, 43] also merit additional research.

Finally, although no publications sufficiently fitted the criteria around sports performance to be included in the targeted review in Table 3, the combination of creatine and caffeine supplements has received some attention. Although these supplements achieve performance benefits via independent mechanisms, previous reports indicated that the simultaneous use of caffeine and creatine (usually in the form of repeated use of both supplements over several days) caused a loss of the ergogenic properties of enhanced phosphocreatine stores [44, 45]. This outcome was attributed to opposing effects of the two supplements on muscle relaxation time [44, 45], although gastrointestinal effects from the acute intake of the combination of products were also reported, separately [46, 47]. However, more recent investigations of chronic creatine supplementation have reported that the acute addition of caffeine prior to a protocol of exercise capacity or performance does not impair the improvements due to caffeine supplementation [48–50]. Although additional sports-specific research on this combination of performance supplements is required, at present there does not seem to be good evidence to prevent athletes from using both of these products in their appropriate scenarios. Furthermore, in general summary on this topic, the combined use of performance supplements is a fertile area of research that requires robust investigation, with a call for careful choice of methodological design to enable clear outcomes to be produced.

## Repeated Use of Supplements

In many sports, competition outcomes are decided through a series of heats and finals, stages in a race or games in a tournament. In other sports, gifted athletes may compete in more than one event in the competition program: for example, Michael Phelps raced 17 times over the 8-day program in which he won eight gold medals at the 2008 Beijing Olympic Games. In some cases, the interval between bouts is measured in hours and may fall within the half-life of a supplement or the body’s return to normal physiological status or homeostasis following the event. Whether the use of an acutely applied supplement known to enhance performance of an event can be repeated for a subsequent event with the same efficacy is therefore of practical importance to competitive sport. At least three different issues might need to be considered in the repeated use of a supplement (see Table 2). The carryover of the first dose to the subsequent event could lead to a modified second protocol, with possibilities ranging from a reduced dose (i.e., if there is still some presence from the first dose that needs to be topped up), to no use (i.e., the supplement is still exerting its full physiological effect in this time frame) as well an increased dose (i.e., if desensitization to the supplement requires a larger amount to achieve the same effect). Whether the use of the supplement for the first event allows a greater physiological effort with undesirable fatigue is also of consideration and may require special pacing strategies for the first event or a decision to use the supplement only for the subsequent (and possibly more important) competition bout.

A thorough literature search, adding the terms “repeated use,” “serial use,” and “subsequent use” to the search terms, was completed to identify sports-specific studies of the previously identified performance supplements that are acutely used (caffeine, bicarbonate, and beetroot juice/nitrate). The case of phosphate loading merits special mention since its application is neither acute (i.e., having a well-established protocol of use targeting a single occasion) nor chronic (i.e., requiring a period of more than several days to achieve the physiological goal and maintaining this status for weeks-months) as is the case for creatine and beta-alanine. Indeed, optimal protocols for the application of phosphate loading to sports performance are still being developed, but at the present time, they consist of 3–5 days of loading with the possible but unconfirmed maintenance of effects for several days [19]. Therefore, there is insufficient information on which suggestions regarding use for repeated competitive events can be based.

The sparse literature on repeated use of well supported performance supplements is summarized in Table 4. Although there is only one specific study regarding the use of caffeine in a simulated two day sporting competition [51], there are several topical themes around serial use of this common dietary ingredient. Previously, the enhancement of exercise capacity associated with caffeine was believed to be reduced by habitual use, necessitating withdrawal from caffeine for several days to achieve a state of caffeine naivety for both the rigor of scientific study and the optimization of competition benefits [15]. An extension of this belief would predict a diminished return for repeated caffeine use in multi-day competitions. Although we now know that caffeine withdrawal does not enhance the magnitude of performance benefits associated with caffeine supplementation [52], the repeated use of caffeine over multi-day sporting events raises other issues. These include the carryover of additional fatigue or muscle damage from the increased effort made possible by caffeine use on the first day, as well as interference with sleep patterns. The effects of performance-related caffeine use on sleep quality and recovery during multi-day competition has not been systematically studied, although it has been identified as a contributor to problems in other sporting situations (e.g., impaired recovery from night matches in team sports [53, 54]). Furthermore, there are anecdotal reports of cyclical use of caffeine and sleeping tablets in some multi-day competitions as athletes seek to counter the effects of each drug [55, 56]. Further research is needed to both identify and investigate such patterns objectively, and to remove the confounding variables that may otherwise affect sleep architecture such as competition arousal, high-intensity exercise and alterations in daily routines. In the meantime, the only available study of repeated use of caffeine in a simulated competition scenario [51] found that modest (3 and 4.5 mg/kg) doses, as recommended in current guidelines for caffeine use in sport, were associated with consistent and significant performance benefits (4–5% enhancement of work done in a cross country skiing time trial) when implemented on two occasions, 24 h apart (Table 4). This benefit occurred despite increased muscle damage and soreness from the first bout undertaken with caffeine, attributed to the greater exercise effort; presumably, the use of caffeine on the second occasions was able to mask these effects [51].

**Table 4:** Effect of repeated use of supplements on sports-related performance protocol

Study	Subjects and design^a^	Supplement protocol	Performance protocol	Enhancement of performance	Summary
Caffeine					
Stadheim et al. [51]	Elite junior cross-country skiers (*n* = 8 M)	3 or 4.5 mg/kg caffeine dose @ 75 min pre event	Cross-country skiing 2 × 10 min x-country skiing TT, separated by 24 h	Event 1: Yes at both doses Event 2: Yes at both doses	Poling distance during the 10-min time-trial was improved with both caffeine doses on day 1 compared with the placebo trial: 4.0 [±3.3] % and 4.0 [±2.9] % for 3 mg and 4.5 mg doses, respectively. Despite greater muscular pain and increased creatine kinase levels on day 2 after caffeine use, performance was improved by 5.0 [±3.6] % and 5.1 [±2.8] %
Beetroot juice/nitrate					
Hoon et al. [57]	Highly-trained cyclists (*n* = 26 M) Crossover design	70-ml (4 mmol nitrate) BJ concentrate @ 2.5 h pre TT1 or @ 2.5 h pre TT2 or 70 ml BJ @ 2.5 pre TT1 + 35 ml BJ top up straight after TT1	Cycling 2 × 4 min cycling TT separated by 75 min (simulation of team pursuit schedule at London Olympic Games)	Event 1: Perhaps Event 2: Potential impairment	Sophisticated mixed model analysis took into account caliber of cyclist and learning/order effect, while identifying 1% improvement as the smallest worthwhile change. Overall, nitrate treatment (acknowledged as a suboptimal dose in light of more recent evidence) was associated with a small but unclear improvement of 1.3 ± 1.7% in the first time trial, but also a potentially unclear impairment of performance in the second time trial, perhaps due to the carryover of additional fatigue [−0.3 ± 1.6%]
Bicarbonate					
Joyce et al. [58]	Highly-trained swimmers (*n* = 8 M) Crossover design	Serial bicarbonate protocol: 3 days @ 300 mg/kg/day sodium bicarbonate divided in 3 daily doses + final dose on day 4 Performance protocol undertaken on days 4 and 5	Swimming 2 × 200 m TT separated by 24 h to simulate swimming competition	Event 1: No Event 2: No	Plasma bicarbonate concentration was increased after 3 days’ serial loading, although to a smaller extent than when same daily amount was taken as acute dose 90 min before the TT. However bicarbonate concentrations returned to baseline 24 h after last dose. There was no enhancement of TT performance immediately after serial loading compared with placebo trial (1:58.53 ± 0:05.64 vs. 1:59.02 ± 0:05.82 and no difference in between this TT and a further TT after 24 h. Mild symptoms of gut discomfort were noted but not different to those noted in acute loading protocol
Carr et al. [37]	Club level rowers (*n* = 4 M, 3 F) Crossover design	Serial bicarbonate protocol: 3 days @ 500 mg/kg/day sodium bicarbonate split into five doses Performance protocol undertaken on days 2 and 4	Rowing 2 × 2000 m TT separated by 48 h to simulate regatta	Event 1: No Event 2: No	Despite the increase in blood bicarbonate, serial bicarbonate supplementation failed to enhance performance in terms of 2000 m mean power, stroke rate or RPE, or characteristics over each 500-m segment. The authors suggest that the lower caliber of rower in this study may explain the small effect size of performance changes. The reliability of performance in response to chronic supplementation protocol was high; suggesting that individual variability in responsiveness can be quickly established from a trial

*BJ* beetroot juice, *M* male, *F* female, *RPE* rating of perceived exertion, *TT* time trial

^a^Randomized, double-blind, placebo-controlled unless noted

The only sports-specific study involving beetroot juice supplementation focused on the schedule for the team pursuit track cycling event on the London Olympic Games Program and involved different protocols that might be implemented for the semi-final and final races, separated by a 75-min interval [57]. One treatment provided beetroot juice concentrate 2.5 h before the first cycling bout (4 min time trial), another delayed it until 2.5 h before the second time-trial, while the third active treatment involved a full dose prior to the first time trial, with a half dose “top up” immediately afterwards (Table 4). A limitation of this study, recognized in light of more recent work, is that the beetroot juice doses would now be considered too low to achieve clear and optimal effects, especially in high caliber athletes [57]. However, it is noted that in comparison to the placebo trial, all beetroot juice trials appeared to provide a small but unclear improvement of the first time-trial but a potential negative effect in the second bout, perhaps due to residual fatigue from the extra demands of the initial effort [57]. Clearly, further work is required around the repeated use of beetroot juice as well as the potential for a carryover effect from performance bouts.

Finally, repeated use of bicarbonate supplementation has been studied in several sports-specific study designs, simulating a successive day use in a swimming carnival [58] or repeated use in a rowing regatta where races may be separated by 48 h [37]. Both studies (see Table 4) used the serial bicarbonate loading protocol in which 300–500 mg/kg of bicarbonate is taken daily in split doses to chronically elevate plasma bicarbonate concentrations [9]. Although the principle underpinning this strategy is to allow the bicarbonate loading protocol to finish the day before competition, reducing the risk of gastrointestinal distress on the day of the event, it may also have relevance to multi-day or repeated events. Both studies failed to detect a positive effect of bicarbonate loading on either of the performance bouts, although issues with the caliber of athletes and the dosing protocol may have interfered with the potential of bicarbonate loading to enhance performance under these conditions. Indeed, a study involving the repetition of acute bicarbonate loading for five consecutive days reported that the benefits to high-intensity cycling were maintained over this period [59]. Therefore, there is merit in further investigation.

## Individual Responses to Supplement Use

The phenomenon of individual responses to supplement use is demonstrated by the life experience that there are two types of people in the world; those who drink coffee after 4 pm and those who would be awake all night if they did. Such (true) individual differences create a few challenges in research and real-life sports nutrition practices. First, they may interfere with the outcomes of a scientific investigation, masking the true potential of the supplement intervention to alter metabolism or performance. Because sport science research traditionally involves small sample sizes, the presence of one or two outliers or contrary responders within a group can render the mean finding from the intervention using probability statistics as a non-significant outcome. This may be compounded by the publication bias or reduced interest in studies that fail to find a clear or positive effect from the applied intervention [60]; preventing the fate of the majority of subjects who reported a potentially useful change from reaching the public domain. A second challenge awaits the expert panels who try to develop guidelines regarding sports nutrition—how definitive can they be when it is apparent that athletes do not all respond in the same way? And finally, there is the concern of each athlete and coach who wants to use their finite time and resources wisely; how can they know even in the case of performance supplements that have received broad scientific support if their own experience will be positive, negative or neutral? A number of important issues in distinguishing real differences in response and characteristics of athletes which might underpin such differences will now be explored.

### Challenges in Determining Real and Meaningful Responses to a Performance Supplement

**Fig. 1:**
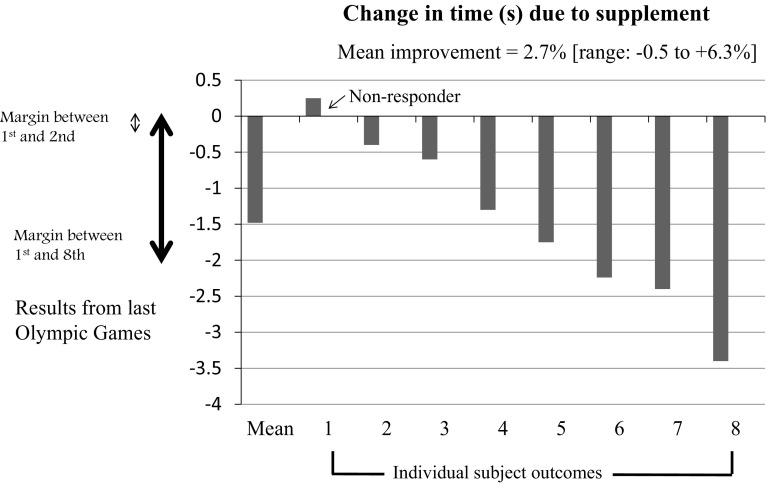
Strategies often used in the presentation and interpretation of results of supplements studies: Identification of individual responses to the intervention, and the comparison of results to the close outcomes often seen in real life sports competition. Both of these devices can lead to a misunderstanding of the real results due to the failure to appreciate the day to day variability in performance of the participants in the study or real-life athletes

Several recent trends in the reporting of studies of performance supplements are of relevance to the current review: the provision of individual results within group data for performance outcomes, and commentary around the significance of these results in terms of typical outcomes of real-life sporting competitions. To demonstrate several common misconceptions around these practices, Fig. 1 provides a fictional example with a simplistic account of the practical implications of the data. It is beyond the scope of this paper to fully review this topic, however, a brief explanation of the problem lies with the failure of researchers (and, presumably, readers) of such analyses to take into account the intrinsic variability in sports performance. The first error in interpretation involves the challenge of distinguishing the change in performance due to the use of the supplement from the normal day to day variability in exercise outcomes. Indeed, in the absence of any other data or context to the reliability of performance measures, it would be unwise to speculate on the cause of any performances differences/changes observed in the study.

The second misunderstanding is that the small inter-competitor differences in real-life sport, i.e., the narrow margins that thrill the spectators at competitive events—set the bar for the magnitude of change that a supplement needs to achieve to be considered effective. It is tempting to think that an athlete needs only to improve by the fractions of seconds or millimeters that frequently determine podium recognition to make the investment in a supplement worthwhile. Yet, as has been eloquently explained by sports statisticians [14], the within-individual differences in sports performance are equally important; these often overlap such that the re-running of many events would likely lead to a different outcome without any intervention being applied.

After modeling the results of a range of sports [14, 61], Hopkins et al. suggest that “worthwhile” changes to the outcome of most events require a performance difference equal to approximately 0.3**–**0.5 times the variability (coefficient of variation [CV]) of performance for that event. Furthermore, across a range of events the CV of performance of top athletes is usually within the range of 0.5–3%, with some variability according to the type of event and the caliber of the performer [61]. However, an improvement of ~0.3–0.7 of the event CV does not guarantee a clear movement of the athlete from finalist to winner; rather, it may simply increase their likelihood of winning by an absolute increase of ~10–20% (for example, improving from a 40% chance of winning to a 50–60% chance) [14]. Such a difference is still outside the realm of detection using probability statistics with the sample sizes normally associated with supplement studies in sports science. Nevertheless, scientists can interpret their results meaningfully by reporting the outcome as a percentage change in the measure of athletic performance and using 90–95% confidence limits to describe the likely range of the true effect of the treatment on the average athlete represented in the study [62]. Despite some dissent on the topic [63], many applied sports scientists find the use of magnitude-based inference techniques, which provide qualitative determinations of the significance of the likely true chance in terms of real-world significance, to be valuable [14, 61, 62, 64].

Now that tools for better examining the overall effect of a performance supplement have been determined, there is opportunity to focus on strategies to differentiate true differences in response to supplements and day to day variability in performance. A logical starting point is consideration of characteristics that might underpin some of the common differences in response.

### Effect of Sex on Responses to Supplements

Males and females differ in many ways that are important for sports nutrition outcomes—for example, differences in body size and body composition, and differences in hormonal profiles that affect metabolism. Although early sports nutrition guidelines featured recommendations in absolute amounts (e.g., an athlete should consume 50 g of carbohydrate after exercise), more recent statements have tried to reflect that a 45-kg female runner may have different needs or responses to a 90-kg male rower. Thus, just as macronutrient targets are now often given relative to body mass (BM) or fat free mass (e.g., an athlete should consume carbohydrate equivalent to 1 g/kg BM after the event), in many cases, doses of supplements are also scaled to body size (e.g., caffeine dose of 3 mg/kg, bicarbonate dose of 300 mg/kg) [4, 9].

Whether the hormonal characteristics of the female athlete and their fluctuations over the menstrual cycle, known to have measureable effects on physiology, create differences in the responsiveness of females to supplement strategies is of interest. In most cases, the literature on sports-specific uses of performance supplements has been largely undertaken with male subjects and research that directly compares male and female responses to interventions is sparse. Although some studies have reported apparent differences in male and female athletes to caffeine supplements [65, 66], issues such as a greater risk of gastrointestinal disturbances during exercise in females and difficulties in matching the athletic caliber and reliability of performance between groups of male and female athletes must also be taken into account in interpreting the results. Despite gaps in the evidence base regarding sex differences with respect to supplement use, we generally believe that female athletes respond similarly to their male counterparts when circumstances are matched.

### Effect of Training Status and Athletic Caliber on Response to Supplements

Most sports nutrition studies are undertaken on participants who range in caliber/training history from recreational to well-trained. Indeed, very few studies are undertaken on world-class athletes. There are suspicions, based on testimonials and some literature, that elite and highly trained athletes may respond differently to some sports nutrition strategies than their lesser trained or less successful counterparts. This issue has been recently examined in relation to beetroot juice/nitrate supplements, where it has been proposed that they may be less effective in enhancing the performance of elite competitors than lower level athletes [67].

As usual, there are issues with the robustness of the available literature that can make it dangerous to make firm conclusions. Nevertheless, there is evidence to support the hypothesis within from single studies in which the benefits of supplementation were inversely correlated with maximal aerobic capacity [68], as well as from a meta-analysis of studies which showed greater effect sizes of performance enhancement with lower caliber athletes [12]. Furthermore, there are credible explanations of how highly competitive sports people might differ from the rest of the athletic population due to the effects of years of training adaptations or the genetic differences that have selected them to be so suited to perform in their event. For example, it has been speculated that compared to moderately trained individuals, elite athletes have a different muscle fiber type composition, greater muscle capillarization and adaptation which reduce the development of hypoxia and metabolic acidosis, and a more developed pathway to produce nitric oxide from arginine; these conditions may reduce the benefit of an enhanced activity of the nitrate-nitrite-nitric oxide pathway [13].

Do these findings mean that elite athletes should not bother to experiment with beetroot juice supplements? A considered cost:benefit analysis finds that many sports scientists would err on the side of the potential benefits [69]. Given that “responders” are still apparent in studies involving elite cohorts and few side-effects other than pink urine/stools following beetroot juice ingestion have been reported, nitrate consumption may still be a useful nutritional strategy for high-caliber athletes [69]. The efficacy of this strategy is likely to be associated with the specific conditions of the sporting event. Shorter, high-intensity events may benefit more due to the greater hypoxic and acidic stress favoring the nitrate-nitrite-nitric oxide pathway and the relatively greater involvement of type II fibers. In particular, the localization of these conditions in smaller muscle groups may favor effects during upper-body dominant exercise [69], explaining observations of benefits to competitive athletes in kayaking and rowing [70, 71]. Applying this strategy to situations of hypoxia, such as altitude training or competition in high altitude environments (e.g., cross-country skiers, mountainous cycling stages) where O_2_ availability is challenged also bears consideration and further study [69]. Therefore, even if there is general evidence that nitrate supplementation is valuable to lesser trained or lower caliber athletes, there are perhaps certain sports, events and conditions where it may assist elite competitors [69].

### True Individual Responses Including Genetic Differences

In any supplementation study, the performance outcomes will include a mixture of results around the mean effect, with the experience of some participants clustering around the “typical” response and other subjects showing a greater or lesser response. Depending on the magnitude and direction of the mean effect, the absolute changes seen among individuals might span from a negative effect through to a very large performance gain. The occurrence of side-effects can also be an individual experience. Although some of these observations can be attributed to random day to day variability in performance which occurs even in the face of attempts to standardize or minimise extraneous variables, there is also the possibility that some of differences are robust and reproducible.

Real differences in responsiveness to a supplement can arise due to a number of factors. These include differences in background nutritional status; for example, creatine loading achieves greater increases in muscle creatine stores in vegetarians than omnivores, associated with their lower starting/baseline stores [72]. Furthermore, caffeine supplementation produces a smaller benefit in endurance protocols in which carbohydrate availability is high (i.e., when carbohydrate is consumed during the exercise protocol compared with placebo/water) [73]. However, there is growing interest in, and awareness of, genetic differences which may also account for variability in responsiveness to a range of interventions. In the general health and nutrition literature, we now appreciate that well-known differences in reactions to the intake of caffeine (an adenosine antagonist) can be explained by differences in the type, number, and site of adenosine receptors around the body, as well as differences in liver metabolism of caffeine which alter its half-life [74]. There is emerging evidence that genetic differences affect caffeine’s ability to enhance sports performance. A study of 36 competitive cyclists subdivided the group according to their expression of a single nucleotide polymorphism of a gene associated with cytochrome P450 (CYP1A2), a family of proteins involved in hepatic metabolism of drugs [75, 76]. Overall, the 16 athletes who were AA homozygotes of this gene achieved a 4.7% improvement in 40-km time (75.1 ± 6.1–71.6 ± 4.3 min, *p* < 0.05), the 19 athletes who carried the C allele achieved only a trend to a performance benefit (73.1 ± 4.5–71.6 ± 4.4 min, non-significant difference). Although this research is in an embryonic phase, it is likely that future work will target the influence of various gene polymorphisms on metabolism of caffeine and other performance supplements and confirm anecdotal observations of differences in the effectiveness of these products.

A final issue in conducting research on performance supplements is to identify techniques that can confirm the presence of individual responsiveness to a sports nutrition intervention. As in all robust research, the starting point is to control study conditions so that the supplement intervention is the only variable that is changing, and to choose or practice a performance outcome to achieve high and known levels of reliability. Such conditions may provide a stable background against which biochemical or physiological markers correlated with the supplement and the change in performance can be clearly detected, and meaningful differences between individuals noted. For example, in a study of creatine supplementation, five of eight subjects showed a meaningful increase in work completed in a repeated sprint cycling test [77]. Examination of muscle substrates identified a significant correlation between the increase in muscle creatine content and the change in work achieved during the cycling test, and showed that only the five “responsive” cyclists achieved a substantial increase in total creatine content as a result of the rapid loading protocol. In a separate study of bicarbonate loading, researchers found that the benefits to high-intensity cycling capacity were associated with both the absence of gastrointestinal symptoms and the magnitude of pre-post exercise changes to plasma pH, base excess and bicarbonate [78]. Studies which include large numbers of subjects and undertake co-variate analysis of various characteristics including genetic profiling or physiological parameters can provide a sophisticated (albeit resource-intensive) opportunity to investigate individual responsiveness.

**Fig. 2:**
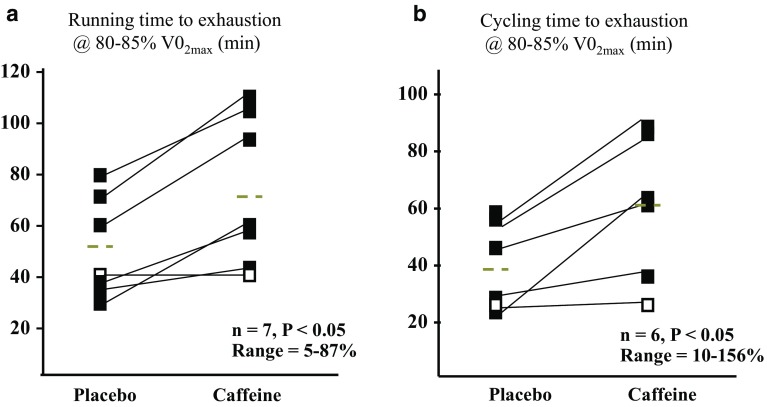
Effects of large doses of caffeine (9 mg/kg) taken 60 min prior to running (**a**) or cycling (**b**) at an intensity equivalent to 85% of maximal aerobic capacity (VO_2_max) on time to exhaustion in a group of seven highly trained runners. Note that one subject (*open square*) is different to the other six subjects (*filled diamond*) in showing a consistently small response to caffeine. Adapted from Graham and Spriet [79], with permission

A simpler strategy is to undertake repeated interventions of the strategy within the trial to investigate the robustness of the response. Studies undertaken by Spriet et al. on caffeine supplementation have achieved this in various ways. An early study of exercise capacity in well-trained runners [79] involved four separate trials in which subjects consumed 9 mg/kg caffeine or a placebo 1 h before either a run or a cycle to exhaustion at 85% of their maximal aerobic capacity (*V*O_2_max). Although the study was focused on effect of caffeine on exercise metabolism and capacity rather than individual responses to this performance supplement, the authors noted that one of the seven subjects showed a consistent and markedly reduced response to the caffeine treatment compared with the other subjects (see Fig. 2). In a more recent study of the effect of low to moderate caffeine doses on performance, well-trained cyclists performed four randomized trials involving placebo, 100 mg, and 200 mg caffeine doses, plus a repeat of one of these trials [80]. Such research design provides confidence in its observations of both the overall benefits of the caffeine treatment as well as any potential differences between subjects.

Within real life practice, high performance athletes who receive sports science support may have access to information about their signature responses to supplement interventions to enable them to identify successful strategies or tweak characteristics. For example, the athlete may be able to track blood pH and bicarbonate levels following bicarbonate loading strategies to optimize the right timing and dose to achieve an ergogenic benefit in a sustained high-intensity event. In the future, it may become possible to receive a genetic profiling report that could identify potential responsiveness to different kinds of strategies. For the moment, the most pragmatic way for an athlete to investigate a sports nutrition intervention is to consider best practice guidelines as a starting point that will cover most individuals. Further to this, they may identify if they are a non-responder or responder to the intervention via trial and error, aided by standardized application and systematic reporting of the outcomes of their activities.

## Conclusion

The use of evidence-based performance supplements may form a small but important part of an athlete’s nutrition plan to maximize the outcomes of training and optimize competition performance. Athletes who are interested to use such supplements are advised to make decisions on several accounts, including the evidence that this will be of specific benefit to their event. However, despite advances in sports nutrition research to improve the methodological rigor of supplement studies, the applicability of such interventions to competitive sport, and the practical interpretation of the results, there are still several issues that remain relatively unexplored. A major real life interest is whether there are additive and/or interactive effects from combining the use of several supplements, based on evidence that each supplement provides a benefit to the event in question when used in isolation. Indeed, there are many justifiable proposals for combinations and permutations of well-supported performance supplements, such as bicarbonate, beta-alanine, creatine, caffeine, nitrate, and phosphate, based on the literature around their individual uses. Some of the potential combinations that could be justified and, indeed, observed in practice within the athletic community, are potentially too complex to be systematically studied in a conventional research design.

The small number of sports-specific studies which have investigated the combination of two of these supplements, in a scenario in which additive benefits might be predicted, have reported a range of outcomes including positive, neutral, or counteractive effects. This range of outcomes may reflect methodological issues (under-powered sample sizes, sub-optimal protocols of supplement use, poor reliability of performance) or differences in the ability of the performance protocol/scenario to achieve a physiological/biochemical challenge of sufficient magnitude to be addressed by the supplements. Further work is needed to add to this body of literature before clear messages can be identified about successful combinations of supplements and when they might be used.

A second issue that has received even less attention arises in sports in which multiple events are carried out within reasonably short periods to decide the final outcome of the event, and in which the athlete might desire to repeat the use of an evidence-based performance supplement. The sparse literature on this issue suggests that there is a range of potential considerations around the successive use of supplements, including overlapping half-lives of the products, the accumulation of fatigue or the desensitization to the effect.

Finally, the issue of individual responsiveness to supplement use represents a challenge both to researchers of studies with small sample sizes and to the athlete who needs to make a decision about whether a product provides a benefit to his/her performance. Part of the bigger challenge of undertaking and interpreting research on sports supplements is to distinguish real and robust effects from the daily variability of performance. However, implementing rigorous standardization protocols and performance measures with high reliability can help to increase the “signal-to-noise” ratio and thus the ability to detect real and worthwhile effects. Other strategies include underpinning observations of performance changes with mechanistic measurements such as physiological or genetic characteristics, or undertaking repeat interventions on the same individual to investigate the consistency of the effect.

In summary, supplement use by athletes is wide-spread and potentially useful to sports performance. Therefore, sports nutrition research should tackle the important questions that athletes and coaches need to consider to make evidence-based decisions about if and how to use a specific product. The use of several supplements in combination and the use of same supplement over successive events provide examples of issues that require further investment with robust and practical research methodologies. Strategies to isolate the variability of benefits to individuals are also a topic for future interrogation.
